# Extracts of Thai *Perilla frutescens* nutlets attenuate tumour necrosis factor-α-activated generation of microparticles, ICAM-1 and IL-6 in human endothelial cells

**DOI:** 10.1042/BSR20192110

**Published:** 2020-05-29

**Authors:** Narisara Paradee, Niramon Utama-ang, Chairat Uthaipibull, John B. Porter, Maciej W. Garbowski, Somdet Srichairatanakool

**Affiliations:** 1 Department of Biochemistry, Faculty of Medicine, Chiang Mai University, Chiang Mai 50200, Thailand; 2 Department of Product Development Technology, Faculty of Agro-industry, Chiang Mai University, Chiang Mai 50200, Thailand; 3 National Center for Genetic Engineering and Biotechnology (BIOTEC), National Science and Technology Development Agency (NSTDA), Pathum Thani 12120, Thailand; 4 Department of Haematology, University College London, Cancer Institute, London WC1E 6BT, United Kingdom

**Keywords:** Perilla frutescens, endothelial cell, inflammation, microparticle, TNF-α, vessel

## Abstract

Elevation of endothelial microparticles (EMPs) play an important role in the progression of inflammation-related vascular diseases such as cardiovascular diseases (CVDs). Thai perilla (*Perilla frutescens*) nutlets are rich in phenolic compounds and flavonoids that exert potent antioxidant and anti-inflammatory effects. We found that the ethyl acetate (EA) and ethanol (Eth) extracts of Thai perilla nutlets contain phenolic compounds such as luteolin, apigenin, chryseoriol and their glycosides, which exhibit antioxidant activity. The goal of the present study was to investigate the effects of the extracts on endothelial activation and EMPs generation in tumour necrosis factor-α (TNF-α)-induced EA.hy926 cells. We found that TNF-α (10 ng/ml) activated EA.hy926 cells and subsequently generated EMPs. Pre-treatment with the extracts significantly attenuated endothelial activation by decreasing the expression of the intracellular adhesion molecule-1 (ICAM-1) in a dose-dependent manner. Only the Eth extract showed protective effects against overproduction of interleukin-6 (IL-6) in the activated cells. Furthermore, the extracts significantly reduced TNF-α-enhanced EMPs generation in a dose-dependent manner. In conclusion, Thai perilla nutlet extracts, especially the Eth extract, may have potential to protect endothelium against vascular inflammation through the inhibition of endothelial activation and the generation of endothelial microparticles (EMPs).

## Introduction

Vascular inflammation plays an important role in the development of cardiovascular diseases (CVDs) such as atherosclerosis [1,2]. Accordingly, proinflammatory cytokines including tumour necrosis factor-α (TNF-α) and interleukin-6 (IL-6) promote endothelial activation through the up-regulation of certain cell adhesion molecules (CAMs), such as the intracellular adhesion molecule-1 (ICAM-1), the vascular cell adhesion molecule-1 (VCAM-1) and E-selectin leading to the recruitment and adhesion of circulating leucocytes, mainly monocytes, to the site of inflammation [3]. Subsequently, the secreted cytokines and chemokines amplify the accumulation of the inflammatory cells and contribute to severe vascular inflammation [4,5]. Indeed, inhibition of endothelial activation is needed to prevent the development of inflammation-related diseases.

Moreover, activated endothelial cells release microparticles (MPs) in response to vascular inflammation. Many stimuli including TNF-α, cytokines, bacterial lipopolysaccharide (LPS) and reactive oxygen species (ROS) can stimulate the release of endothelial microparticles (EMPs) [6,7]. EMPs (<1 µm in diameter) are shed from the plasma membranes of endothelial cells, carrying proteins such as ICAM-1, and expose the anionic phosphatidylserine (PS) at their surface. Many studies have revealed that EMPs promote endothelial activation, coagulation and thrombosis through their PS membranes leading to the acceleration of the progression of vascular inflammation [8]. In particular, EMPs are considered a potential biomarker of endothelial dysfunction in CVDs [9]. TNF-α can induce oxidative stress and reduce nitric oxide (NO^•^) production resulting in an increase in the EMPs generation and the impaired function of endothelial (EA.hy926) cells [4].

*Perilla frutescens* (L.) Britton fruits have long been consumed medicinally and as a functional food in East Asian countries, including Thailand. Perilla nutlets are rich in α-linolenic acid (ALA) and contain phenolic compounds and flavonoids that exert antioxidant, anti-allergic, anti-cancer and anti-inflammatory effects [10,11]. Interestingly, the ethanol extract of perilla seeds was found to reduce levels of NO^•^, IL-6 and TNF-α in LPS-stimulated RAW264.7 macrophages [12]. Importantly, ethyl acetate and the ethanol extracts of Thai perilla nutlets containing the flavones apigenin, luteolin and chrysoeriol exhibit antioxidant and hepatic anti-lipid peroxidation activities [13]. However, the effects of Thai perilla fruits on cytokine-induced endothelial cell activation are less well known. The aim of the present study was to determine the effects of Thai perilla nutlet extracts on endothelial activation and EMPs production in TNF-α induced EA.hy926 cells.

## Materials and methods

### Chemicals and reagents

Calcein-acetomethoxy (Calcein-AM), CountBright™ absolute counting beads, 2′,7′–dichlorofluorescin diacetate (DCFH-DA), Dulbecco’s modified Eagle’s medium (DMEM), fetal bovine serum (FBS), human TNF-α, penicillin–streptomycin, RPMI 1640 and trypsin-EDTA were purchased from Thermo Fisher Scientific, Inc., Waltham, MA, U.S.A. Fluorescein isothiocyanate-Annexin V (FITC-Annexin V) and phycoerythrin (PE)-mouse anti-human CD54 were purchased from BD Biosciences, San Jose, CA, U.S.A. Additionally, 3-(4,5- Dimethylthiazol-2-yl)-2,5-diphenyltetrazolium bromide (MTT) and phosphate-buffered saline (PBS) at a pH of 7.0 were purchased from Sigma–Aldrich Inc., St. Louis, MO, U.S.A. An enzyme-linked immunosorbent assay (ELISA) kit for human IL-6 was purchased from Boster Biological Technology, Pleasanton, CA, U.S.A.

### Preparation of perilla nutlet extracts

Thai perilla (*P. frutescens*) nutlets were harvested from Wienghang District, Chiang Mai Province, Thailand in 2014 and were botanically examined by Dr. C. Leon, Royal Botanic Gardens (RBG) Kew, Richmond, United Kingdom. These nutlets were compared botanically and chemically with authentic *P. frutescens* nutlets from the Royal Botanic Gardens Kew Economic Botany Collections, voucher reference: EBC 81840 TCMK 411. The nutlets were pressed to release oil and produce a residue, which sequential extracted according to our recently established method [13]. Briefly, the nutlet residue (500 g) was initially extracted twice with ethyl acetate (EA) at room temperature for 7 days. The EA extracts were combined and concentrated using a rotatory evaporator. The residue following the EA extraction was sequentially extracted with 80% ethanol (Eth) at room temperature for 7 days. Finally, the Eth extracts were combined and dried by rotary evaporation and lyophilisation. The EA extract (10% yield, *w/w*) containing apigenin, luteolin, chryseoriol and their glycosides, revealed total phenolic and flavonoid contents at 6.65 ± 0.05 mg gallic acid equivalent (GAE)/g and 4.52 ± 0.11 mg quercetin equivalent (QE)/g, respectively. In addition, the Eth extract (5% yield, *w/w*) containing rosmarinic acid, apigenin, luteolin, chryseoriol and their glycosides, revealed total phenolic and flavonoid contents at 70.77 ± 0.37 mg GAE/g and 19.97 ± 0.36 mg QE/g, respectively. The extracts were reconstituted in DMSO and diluted with culture medium at designated concentrations in the experiments.

### Cell culture

Human vascular endothelial (EA.hy926) cells were cultured in DMEM supplemented with 10% FBS and 100 IU/ml penicillin and streptomycin in a humidified 5% CO_2_ incubator at 37°C [4]. Human promyelocytic leukaemia (HL-60) cells were grown in RPMI 1640 medium that was supplemented with 10% FBS and 100 IU/ml penicillin and streptomycin in a humidified 5% CO_2_ incubator at 37°C [14].

### Cell viability assay

Cell viability were enumerated based on the mitochondrial reducing power of living cells that could reduce the MTT substrate to a blue-coloured formazan product [14]. Briefly, EA and Eth extracts were solubilised with DMSO to achieve various concentrations. Consequently, EA.hy926 cells were treated with the extracts (50–400 µg/ml) at 37°C for 24 h. After incubation, the treated cells were incubated with MTT solution (5 mg/ml) for 4 h. The formazan crystals were then dissolved with DMSO and the optical density was measured at 570 nm using a 96-well microplate reader.

### Determination of intracellular ROS production

Intracellular ROS production was measured by dichlorofluorescein (DCF) assay [15]. Briefly, EA.hy926 cells were incubated with DCFH-DA (10 μM) at 37 °C for 30 min. After incubation, the cells were washed with PBS and treated with ascorbic acid (200 µg/ml) or the extracts (50–200 µg/ml) for 1 h. The cells were then stimulated with TNF-α (10 ng/ml) for another 24 h. After stimulation, fluorescent intensity (FI) was measured using a fluorescence microplate reader at an excitation wavelength of 485 nm and an emission wavelength of 535 nm. The results were expressed as percentage of fluorescence intensity relative to the control cells.

### Detection of ICAM-1 expression

ICAM-1 plays an important role in mediating leucocyte tethering by rolling on the surface of the vascular endothelial cells. We used anti-CD54 antibody to assess ICAM-1 expression and flow cytometry to detect the red FI of PE [16]. Briefly, EA.hy926 cells were pre-treated with EA and Eth extracts (50–200 µg/ml) for 1 h and were stimulated with TNF-α (10 ng/ml) for 4 h. Cells were then collected and stained with PE–conjugated anti-CD54 antibody, which had previously been prepared in the complete medium for 30 min on ice. After incubation, the cells were washed twice with PBS and analysed (10000 cell events) using a flow cytometer (Beckman Coulter CyAn ADP, Brea, CA, U.S.A.) at 488 nm excitation and 570 nm emission wavelengths. Data were analysed using Kaluza 1.5a software. Fluorescent histogram was set to identify CD54 positive. ICAM-1 expression was expressed as mean FI.

### Evaluation of HL-60 cell adhesion to EA.hy926 cells

The binding of human promyelocytic leukaemia cells (HL-60 cells) to EA.hy926 cells was determined using the previously described methods [3,17]. Briefly, EA.hy926 cells were pre-treated with EA and Eth extracts (50–200 µg/ml) at 37°C for 1 h, stimulated with TNF-α (10 ng/ml) for 4 h and washed with a serum-free medium. HL-60 cells labelled with calcein-AM (1 × 10^5^ cells/well) were then co-cultured with the treated EA.hy926 cells for 30 min at 37°C. Non-adherent HL-60 cells were then removed by washing the cells three times with PBS. Mean FI of adherent HL-60 cells was measured with a 96-well plate spectrofluorometer (485 nm excitation wavelength/530 nm emission wavelength), and their fluorescent imaging was observed by employing the High-Content Analysis System.

### Measurement of IL-6 levels using ELISA kit

IL-6 production was measured by commercial enzyme-linked immunosorbent assay (ELISA) kit. Briefly, EA.hy926 cells were pre-treated with EA and Eth extracts (50–200 µg/ml) for 1 h and then treated with TNF-α (10 ng/ml) for 24 h at 37°C. Afterwards, the culture medium was collected and centrifuged at 1500 rpm for 10 min at 4°C. Finally, the supernatant was quantified for IL-6 using an ELISA kit according to the manufacturer’s instructions.

### Flow-cytometric analysis of EMPs

Amounts of EMPs were measured using the flow cytometry method [4,18]. In the assay, EA.hy926 cells were pre-treated with EA and Eth extracts (50–200 µg/ml) for 1 h and incubated with TNF-α (10 ng/ml) for 24 h. After incubation, the culture medium was collected and centrifuged at 5000×***g*** for 15 min. The supernatant was collected and centrifuged at 20000×***g*** for 40 min. The supernatant was discarded, and the EMPs pellet was reconstituted in Annexin V binding buffer and stained with FITC-Annexin V for 15 min in the dark at room temperature. Subsequently, CountBright™ counting beads were added in each sample as an internal control and 5000 counting bead events were analysed using FACSCalibur Flow Cytometer (BD) with BD CellQuest Pro Software (BD Biosciences, San Jose, CA, U.S.A.). For EMPs gating, calibrated latex beads (1.1 µm diameter) were used to set the population of particle size < 1 µm. The gated populations which stained with FITC-Annexin V were used to set the subpopulation of positive Annexin V EMPs. The numbers of positive Annexin V EMPs were normalised with the counting beads and were presented as absolute numbers of EMPs as well as the fold-change of EMPs relative to the control.

### Statistical analysis

Data are presented as mean ± SEM values. Statistical significance was analysed using one-way analysis of variance (ANOVA) with post hoc Tukey–Kramer’s test, for which *P*<0.05 was considered significant.

## Results

### Cytotoxic effect of perilla nutlet extracts

**Figure 1:**
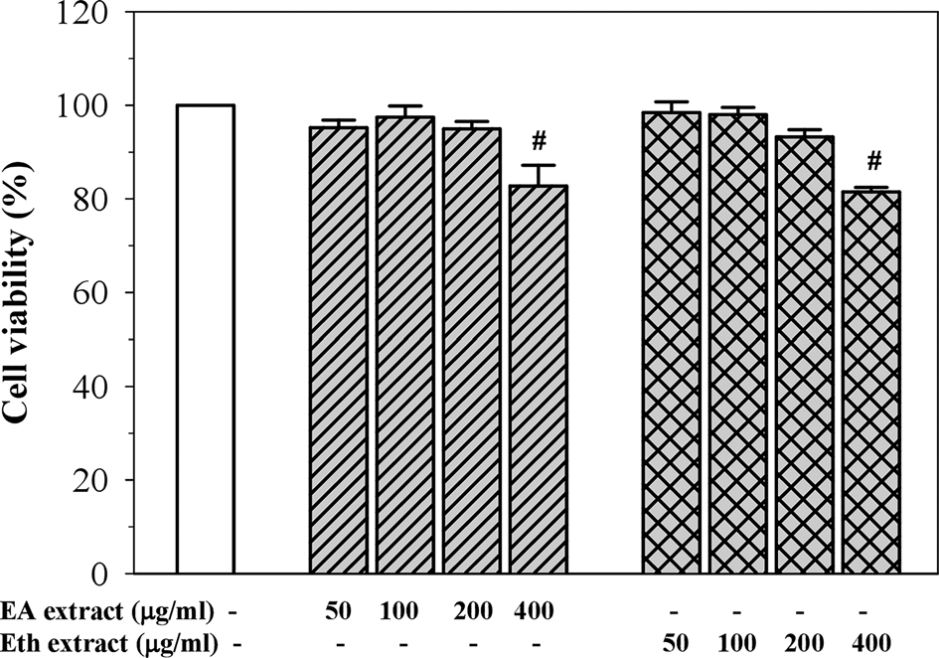
Cell viability after treatment with EA and Eth extracts in EA.hy926 cells Data obtained from three independent experiments are expressed as mean ± SEM. ^#^*P*<0.05 when compared with treatment without the extracts.

As shown in Figure 1, EA and Eth extracts at concentrations of 50–200 µg/ml were not found to be toxic to EA.hy926 cells (cell viability > 90%). As doses of the extracts were raised to 400 µg/ml, the number of viable cells were found to significantly decrease when compared with the control group (approximately 80% cell viability). Based on these results, further experiments were performed using the extracts at a concentration of 50–200 μg/ml.

### Inhibition of intracellular ROS production

**Figure 2:**
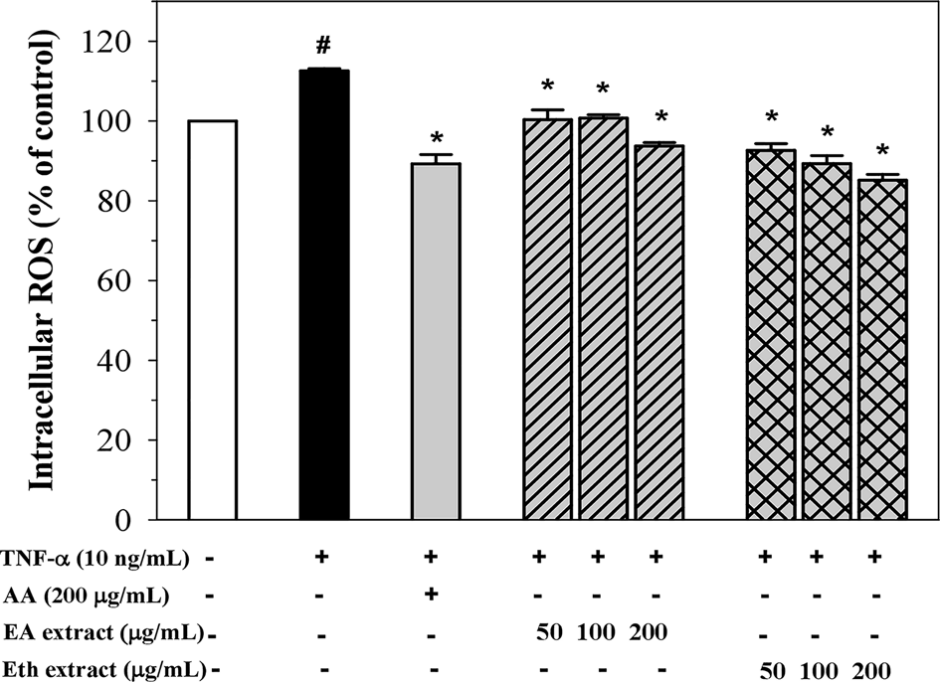
Levels of intracellular ROS production in TNF-α-induced EA.hy926 cells treated with ascorbic acid (AA), EA and Eth extracts Data obtained from three independent triplicate experiments are expressed as mean ± SEM. ^#^*P*<0.05 when compared with nontreatment; *P<0.05 when compared with TNF-α-induced cells.

Stimulation of EA.hy926 cells with TNF-α (10 ng/ml) for 24 h significantly increased intracellular ROS production (Figure 2). Pre-treatment with ascorbic acid (200 µg/ml) significantly decreased the production of intracellular ROS. Indeed, both EA and Eth extracts significantly reduced the generation of intracellular ROS in a dose-dependent manner, where the Eth extracts were found to be more potent than those of the EA extract.

### Inhibition of ICAM-1 expression

**Figure 3:**
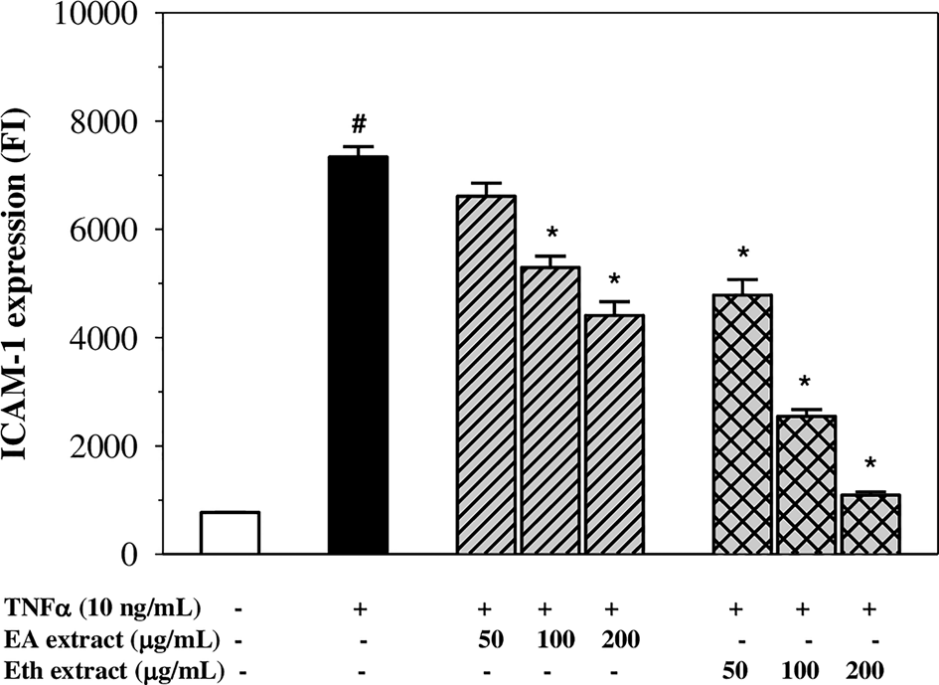
Levels of ICAM-1 expression in TNF-α-induced EA.hy926 cells treated with EA and Eth extracts Data obtained from three independent experiments are expressed as mean ± SEM. ^#^*P*<0.05 when compared with nontreatment; *P<0.05 when compared with TNF-α-induced cells.

Exposure of EA.hy926 cells to TNF-α (10 ng/ml) for 24 h exhibited no cytotoxicity, whereas the exposure for 4 h was found to elevate the expression of ICAM-1 on the EA.hy926 cell membrane when compared with the control (*P*<0.05) (Figure 3). Pre-treatment of the cells with Eth extract (50–200 µg/ml) significantly reduced the expression of ICAM-1 in a concentration-dependent manner. Similarly, the EA extract decreased the expression of ICAM-1 in a dose-dependent manner with 100–200 µg/ml presenting significant inhibitory effects (decreasing expression to 30 and 15% of control, respectively). The results revealed that the EA and Eth extracts exhibited anti-inflammatory effects through the inhibition of adhesion molecule expression on TNF-α-stimulated endothelial cells.

### Effects on adhesion of HL-60 cells to TNF-α-activated EA.hy926 cells

**Figure 4:**
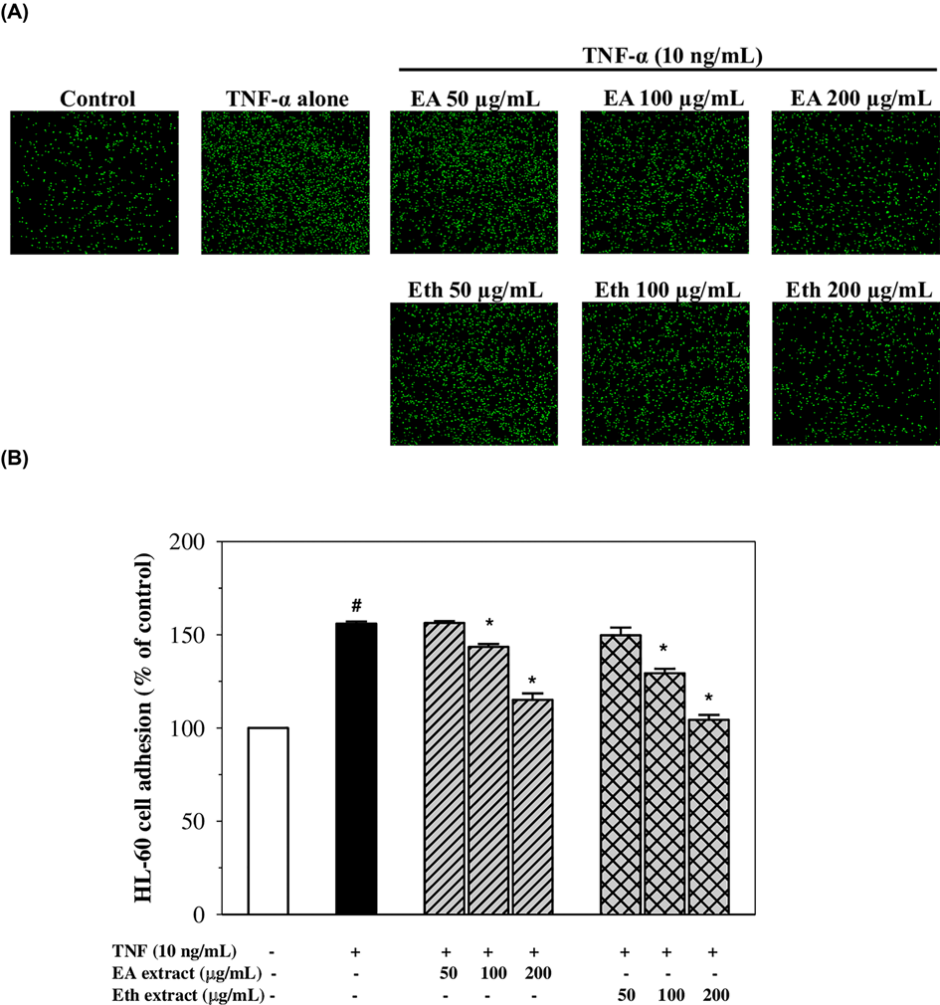
Adhesion of HL-60 cells to TNF-α-activated EA.hy926 cells after treatment with EA and Eth extracts Fluorescent signal (**A**) and percentage (**B**) of HL-60 cells adhering to the TNF-α- activated EA.hy926 cells treated with EA and Eth extracts. Data obtained from three independent experiments are expressed as mean ± SEM. ^#^*P*<0.05 when compared with nontreatment; *P<0.05 when compared with TNF-α-induced cells.

Clearly, TNF-α significantly induced adhesion of HL-60 cells to EA.hy926 cells by 1.56-fold when compared with the control (*P*<0.05). Indeed, the adhesion was decreased by pre-treatment with the EA and Eth extracts in a concentration-dependent manner with significant effects observed at concentrations of 100–200 µg/ml (decreasing adhesion to approximately 75 and 30% of TNF-α control for the EA extract, and 60 and 10% of TNF-α control for the Eth extract, respectively) (Figure 4). This broadly confirms the effects of the extracts on the ICAM-1 expression described above. The results suggest that the EA and Eth extracts could protect against the adhesion of leucocytes to vascular endothelial cells as a consequence of their anti-inflammatory effect.

### Inhibition of IL-6 secretion

**Figure 5:**
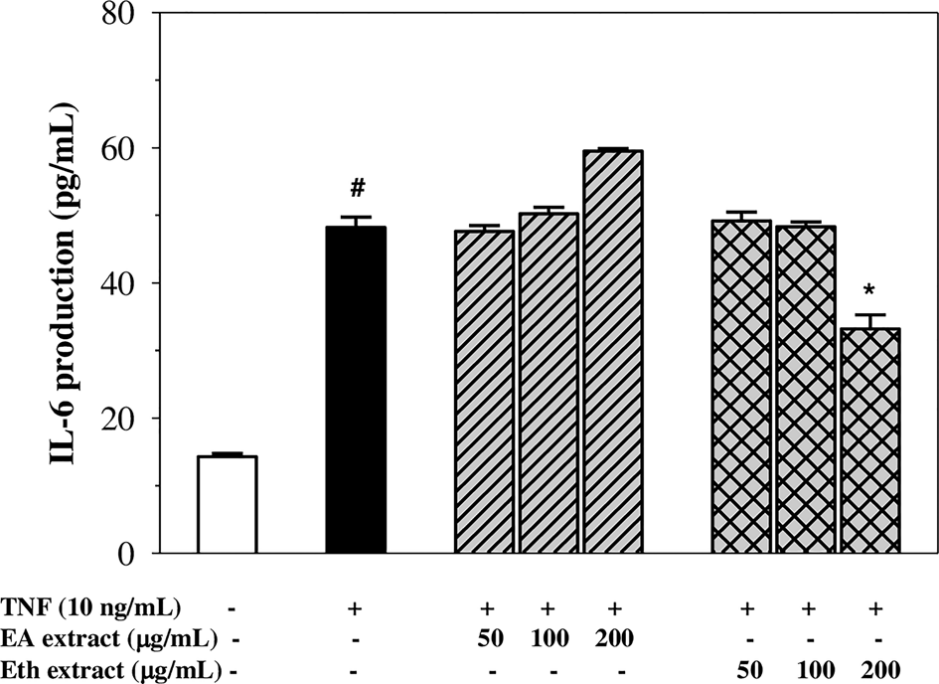
Levels of IL-6 production in TNF-α-activated EA.hy926 cells treated with EA and Eth extracts Data obtained from three independent experiments are expressed as mean ± SEM. ^#^*P*<0.05 when compared with nontreatment; *P<0.05 when compared with TNF-α-induced cells.

As shown in Figure 5, exposure of EA.hy926 cells to TNF-α showed approximately a 3-fold increase in IL-6 secretion when compared with the control (*P*<0.05). Pre-treatment of the cells with the Eth extract suppressed IL-6 production; however, only a dose of 200 µg/ml presented a significant effect. In comparison, pre-treatment with the EA extract did not inhibit TNF-α-induced IL-6 secretion in the EA.hy926 cells. This result implies that the Eth extract, but not the EA extract, may exert the anti-inflammatory effect of reducing IL-6 production in TNF-α-activated EA.hy926 cells.

### Inhibition of EMPs generation

**Figure 6:**
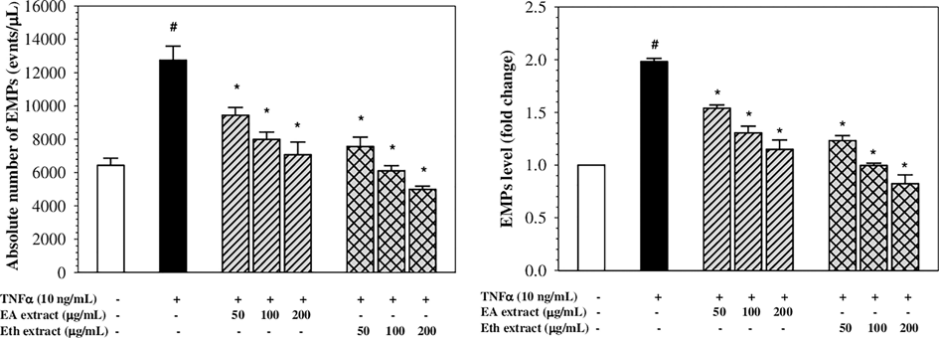
Generation of EMPs in TNF-α-induced EA.hy926 cells treated with EA and Eth extracts Data obtained from three independent experiments are expressed in absolute numbers and fold change as mean ± SEM. ^#^*P*<0.05 when compared with nontreatment; *P<0.05 when compared with TNF-α-induced cells.

Absolute numbers and fold changes of Annexin V+ EMPs are shown in Figure 6. Interestingly, TNF-α (10 ng/ml) was found to increase levels of EMPs generation by 1.98 ± 0.05-fold in EA.hy926 cells when compared with the control (*P*<0.05). Pre-treatment with EA and Eth extracts (50–200 µg/ml) significantly decreased the levels of EMPs generation in a concentration-dependent manner in TNF-α-induced EA.hy926 cells. The Eth extract was found to be more potent than the EA extract, in which the Eth extract at 100-200 µg/ml completely abrogated TNF-α-induced EMPs generation. The results imply that EA and Eth extracts may protect endothelial cells from TNF-α-induced EMPs generation.

## Discussion

Previous studies have demonstrated that perilla fruits are rich in phenolic compounds including flavonoids that exhibit antioxidant and anti-inflammatory activities [10–12]. Recently, we have reported that the flavones such as apigenin, luteolin and chrysoeriol, and their glycosides were detected in the EA and Eth extracts of Thai perilla nutlets [13]. However, rosmarinic acid and its glycoside were found only in the Eth extract [13]. Moreover, the Eth extract revealed higher phenolic and flavonoid contents, higher compound diversity and greater antioxidant activity than the EA extract [13]. In the present study, the EA and Eth extracts in doses up to 200 μg/ml were found to be nontoxic (cell viability > 90%) to human endothelial (EA.hy926) cells but were significantly toxic at 400 μg/ml. Consistently, we have shown in our previous study that both extracts at doses up to 200 μg/ml were nontoxic to human hepatocellular carcinoma (Huh7) cells and expressed cytotoxicity at higher doses. According to the findings of our previous study, both extracts inhibited intracellular ROS in FeCl_3_-induced HuH7 cells. In this study, we also evaluated the antioxidant activity of the extracts by assessing their ability to inhibit intracellular ROS production in TNF-α-induced EA.hy926 cells. TNF-α is one of the stimulators of ROS generation that can cause cellular damage as well as inflammation. Our results revealed that the EA and Eth extracts significantly inhibited the generation of ROS in TNF-α-induced EA.hy926 cells in a concentration-dependent manner. However, only the Eth extracts at a dose of 200 μg/mL displayed more potency in terms of antioxidant activity than those of ascorbic acid, which is known as a standard antioxidant. The Eth extract also had more potency than those of the EA extract, which are in accordance with those of our previous study [13]. It is possible that the anti-oxidant activity of the perilla extracts depended upon the detected compounds and contents of phenolics and flavonoids. These findings suggest that antioxidants contained in both extracts could prevent oxidative stress in human endothelial cells.

In the present study, the EA and Eth extracts exhibited anti-inflammatory activity through the inhibition of endothelial activation in TNF-α-induced EA.hy926 cells. Notably, the Eth extract was found to be more potent than the EA extract. It is possible that the anti-inflammatory effect may occur in accordance with their phenolic and flavonoid contents and their respective free-radical scavenging activity. Phenolic compounds have been reported to display anti-inflammatory activity in both *in vitro* and *in vivo* experiments [19–21]. During the inflammation response, ROS are generated from activated inflammatory cells, resulting in oxidative stress that can amplify the damage to the tissues. Thus, phenolic compounds including flavonoids are desirable in inhibiting the progression of inflammation due to their antioxidant activity and anti-inflammatory activity. In particular, rosmarinic acid, mostly existing in rosemary and ethanolic extracts obtained from plants, was found to exert anti-inflammatory effects on cells, possibly through inactivation of enzymes such as 5-lipoxygenase, inducible nitric oxide synthase and cyclooxygenase-2 (COX2), and signal proteins such as TNF-α, mitogen-activated protein kinases (MAPKs), nuclear factor kapper B (NF-kB), E-cadherin, vimentin, p38, c-Jun NH_2_-terminal kinase 1/2 (JNK1/2) and Janus kinase 2 (JAK2), as well as signal transducers and activators of transcription 1/3 (STAT1/3) [22–24]. Interestingly, previous studies found that perilla extract or rosmarinic acid obviously decreased the degree of gene expression of ICAM-1 and COX-2 in 12-tetradecanoylphorbol 13-acetate treated animals, suggesting anti-inflammatory and ROS scavenging effects [25,26].

During inflammation, vascular endothelial cells become activated by TNF-α that is secreted from immune cells (e.g. macrophages, monocytes, mast cells, T cells and natural killer cells) and ultimately respond to express ICAM-1 molecules. Accordingly, ICAM-1 will be expressed on the surface of the endothelial cells and function to mediate an interaction between the activated endothelial cells and leucocytes, leading to infiltration of the immune cells [1,27]. Moreover, the aberrant expression of ICAM-1 is associated with other inflammatory diseases such as atherosclerosis, arthritis and cancer [28,29]. In the present study, we have found that the Eth extract containing rosmarinic acid, apigenin and luteolin revealed more potent anti-inflammatory effects than the EA extract containing only apigenin and luteolin. These findings are also in accordance with the suppression of ICAM-1 expression by the EA and Eth extracts, in which rosmarinic acid in the Eth extract may cooperate with apigenin and luteolin in the inhibition of ICAM-1 expression and the amelioration of TNF-α-induced inflammation. Evidently, rosmarinic acid of *Perilla frutescent* leaves displayed anti-inflammatory effects via the potent inhibition of TNF-α-induced endothelial protein C receptor shedding by suppression of the TNF-α-converting enzyme [30]. Previous study found that polyphenols, including rosmarinic acid, caffeic acid, ferulic acid and quercetin, were present in the butanol extract of mullein (*Verbascum phlomoides* L.) flowers and were found to exert anti-inflammatory action by inhibiting ICAM-1 expression on TNF-α-induced human umbilical vein endothelial cells (HUVECs) [26]. In addition, apigenin was found to be an anti-allergic inflammatory agent by decreasing the degree of expression of ICAM-1 and IL-6 within the mRNA and protein levels in di-(2-ethylhexyl) phthalate (DEHP)-stimulated HUVECs [31]. Notably, luteolin inhibited ICAM-1 expression in TNF-α-induced cells and mice [3,32,33]. Moreover, rosmarinic acid suppressed ICAM-1 expression in TNF-α-stimulated human fibroblast cells [34,35].

Here, we have documented that EA and Eth extracts were able to potently block the adhesion of HL-60 cells to TNF-α-stimulated EA.hy926 cells in a concentration-dependent manner with even higher inhibitory effects being observed in the Eth extract. In previous studies, apigenin was found to reduce the binding of monocytes to DEHP-induced HUVECs [31] and luteolin suppressed adhesion of monocytes to TNF-α-induced EA.hy926 cells and HUVECs [3]. In these findings, the bioactive compounds present in the EA and Eth extracts may exert an anti-inflammatory effect by inhibiting the interaction between endothelial cells and leucocytes, consequently resulting in a decrease in CAM expression on the endothelial cell membrane.

Pro-inflammatory cytokines such as IL-6 are key mediators involved in enhancing vascular inflammation [36]. The release of IL-6 from activated endothelial cells results in the activation of the immune system through the recruitment of leucocytes and by promoting neutrophil apoptosis [37,38]. Our results have shown that the Eth extract decreased the production of IL-6 in TNF-α-induced EA.hy926 cells with a significant effect occurring only at a dose of 200 µg/ml. However, previous studies have revealed that apigenin inhibited the secretion of IL-6 in THP-1-derived macrophages and compound 48/80-induced mice [31,39], whereas luteolin reduced the release of IL-6 and TNF-α in LPS-stimulated RAW264.7 cells [40]. Indeed, rosmarinic acid reduced the production of IL-6 in LPS-induced acute lung injuries and dextran sulphate-induced colitis in mice [41,42]. Our observations have identified the anti-inflammatory effects of the polyphenolics that are present in Eth extract in the inhibition of IL-6 production.

It has been widely documented that EMPs are involved in the regulation of inflammation, vascular injury and thrombosis [6,9]. Similar findings have demonstrated that EMPs exerted pro-coagulant activity through their PS-rich outer membrane leaflet, which was bound to coagulation factors II, Va and Xa, and subsequently initiated thrombosis [7]. EMPs can promote vascular inflammation through their endothelial adhesion molecules leading to the recruitment of inflammatory cells [2,6]. In our present study, TNF-α (10 ng/ml) significantly induced the release of EMPs in EA.hy926 cells; however, pre-treatment of the cells with EA and Eth extracts potently inhibited the TNF-α-induced generation of EMPs in a dose-dependent manner. These results confirm the anti-inflammatory effects of the EA and Eth extracts of perilla nutlets on TNF-α-induced endothelial cell activation, the inhibitory effects on EMPs generation and attenuation in the severity of endothelial activation and vascular inflammation-related diseases.

## Conclusions

Thai perilla nutlet extracts, especially the Eth extract containing rosmarinic acid, apigenin and luteolin, potentially protect endothelial cell activation via inhibition of ICAM-1 expression and IL-6 production, adherence of leucocytes to endothelial cells, and EMPs generation. Conclusively, these events indicate the relevant inhibitory effects of Thai perilla nutlet extracts on vascular inflammation and CVDs.

## Abbreviations

calcein-AM: calcein-acetomethoxy
CAM: cell adhesion molecule
CVD: cardiovascular disease
DEHP: di-(2-ethylhexyl) phthalate
DMEM: Dulbecco’s modified Eagle’s medium
EA: ethyl acetate
ELISA: enzyme-linked immunosorbent assay
EMP: endothelial microparticle
Eth: ethanol
FBS: fetal bovine serum
FI: fluorescent intensity
FITC: fluorescein isothiocyanate
GAE: gallic acid equivalent
HL-60 cell: human promyelocytic leukaemia cell
HUVEC: human umbilical vein endothelial cell
IC_50_: inhibitory concentration at 50%
ICAM-1: intracellular adhesion molecule-1
IL: interleukin
LPS: lipopolysaccharide
MP: microparticle
MTT: 3-(4,5- dimethylthiazol-2-yl)-2,5-diphenyltetrazolium bromide
NO^•^: nitric oxide
PBS: phosphate-buffered saline
PE: phycoerythrin
PS: phosphatidylserine
QE: quercetin equivalent
ROS: reactive oxygen species
TNF-α: tumour necrosis factor-α
